# 1,2-Dicinnamoyl-*sn*-glycero-3-phosphocholine Improves Insulin Sensitivity and Upregulates mtDNA-Encoded Genes in Insulin-Resistant 3T3-L1 Adipocytes: A Preliminary Study

**DOI:** 10.3390/nu16183163

**Published:** 2024-09-19

**Authors:** Aneta Cierzniak, Anna Gliszczyńska, Małgorzata Małodobra-Mazur

**Affiliations:** 1 Department of Forensic Medicine, Division of Molecular Techniques, Wroclaw Medical University, Sklodowskiej-Curie 52, 50-369 Wrocław, Poland; aneta.cierzniak@umw.edu.pl; 2 Department of Food Chemistry and Biocatalysis, Wrocław University of Environmental and Life Sciences, Norwida 25, 50-375 Wrocław, Poland

**Keywords:** insulin resistance, cinnamic acid, phospholipid derivative, phenolic acid, 1,2-di-CA-PC

## Abstract

Background: Insulin resistance is a condition characterized by a reduced biological response to insulin. It is one of the most common metabolic diseases in modern civilization. Numerous natural substances have a positive effect on metabolism and energy homeostasis including restoring the proper sensitivity to insulin. There may be several possible mechanisms of action. In the present study, we elucidated two natural compounds with an impact on insulin signaling in IR adipocytes involving mitochondria. Methods: Mature 3T3-L1 adipocytes with artificially induced insulin resistance by palmitic acid (16:0) were used for the study. Cinnamic acid and 1,2-dicinnamoyl-*sn*-glycero-3-phosphocholin (1,2-diCA-PC) were tested at three concentrations: 25 μM, 50 μM, and 125 μM. The number of mitochondria and the expression of genes encoded by mtDNA were elucidated in control and experimental cells. Results: Experimental cells treated with 1,2-diCA-PC displayed increased insulin-stimulated glucose uptake in a dose-dependent manner, accompanied by an increase in mtDNA copy number. Moreover, in experimental cells treated with 1,2-diCA-PC at a concentration of 125 μM, a significant increase in the expression level of all analyzed genes encoded by mtDNA compared to control cells was observed. Our study showed a relationship between improved cellular sensitivity to insulin by 1,2-diCA-PC and an increase in the number of mitochondria and expression levels of genes encoded by mtDNA. Conclusions: To summarize, the results suggest the therapeutic potential of cinnamic acid derivative 1,2-diCA-PC to enhance the insulin sensitivity of adipocytes.

## 1. Introduction

Insulin is a key hormone regulating energy homeostasis and metabolic processes within the human body, primarily affecting adipose tissue, skeletal muscle, and the liver [1]. Insulin resistance (IR) is a major public health problem worldwide. It is a condition characterized by a reduced biological response of peripheral tissues to insulin and generally occurs in conjunction with numerous metabolic disorders as well as hormonal disorders such as polycystic ovary syndrome (PCOS) and thyroid dysfunction [2]. A common pathological feature in various forms of IR is the disruption of cellular insulin signal transmission at the molecular level, which is responsible for the development of IR. The main mechanism of insulin resistance induction is connected with impairments in insulin signal transduction, primarily through Akt and phosphatidylinositol 3 (PI3) kinases, which mediate the appropriate effects in the aforementioned tissues. Furthermore, IR induces hepatic gluconeogenesis, which further increases glycemia [3].

Additionally other mechanisms are implicated in the development of IR. Disruption in lipid metabolism is the most common cause of IR development. Moreover, obesity, chronic low-grade inflammation in various tissues, hormonal dysregulation, and other factors also impair proper signal transduction, ultimately leading to disturbances in the mechanisms of insulin action [3]. Generally, lipid metabolism and the inflammatory pathway are intertwined processes which can lead to development of insulin resistance and other metabolic disorders. One of the most important links between lipids, inflammation, and insulin resistance is ceramide biosynthesis [4]. Obesity, besides the elevated lipid accumulation in adipose tissue, is characterized by lipid accumulation in other organs and elevated level of plasma free fatty acids (FFAs). On the other hand, the increase in FFAs in obesity is linked with the activation of Toll-like receptor 4 (TLR4) signaling in adipocytes, which induces the inflammatory process [5]. Furthermore, FFA excess affects mitochondria function by producing ROS, increasing mitochondrial proton conductance, and provoking the permeabilization of the outer mitochondrial membrane [6].

Mitochondria play a crucial role in cell metabolism and are responsible for controlling numerous cellular processes that require ATP. It has also been shown that in humans and other organisms, IR strongly correlates with reduced mitochondrial function, manifested by a reduced mitochondrial copy number, reduced mitochondrial biogenesis, and disruption in oxidative phosphorylation. Moreover, IR leads to impaired energy production in the form of ATP, caused by reduced mitochondrial respiratory capacity observed in insulin-resistant patients, type 2 diabetic patients, and obese individuals [7,8]. The main consequence of reduced mitochondrial capacity is an increase in reactive oxygen species (ROS) production, which is believed to be the root cause of IR development [9].

The current treatment of IR involves several pharmaceutics of which metformin is the first choice of pharmacotherapy. Unfortunately, it is not effective for some individuals; on the other hand, other patients suffer from numerous side effects. Numerous natural compounds have been shown to be effective agents in IR treatment, including various phenolic acids.

Cinnamic acid belongs to the class of aromatic acids and is a member of the phenylpropanoid family of natural compounds. It occurs naturally in various plants and essential oils and is an important intermediate in the biosynthesis of many secondary metabolites. Cinnamic acid exhibits various biological activities, including antioxidant and anti-inflammatory properties, and modulates glycogenesis and gluconeogenesis [10,11]. It has been observed that cinnamic acid exhibits antidiabetic effects by improving glucose tolerance and stimulating insulin secretion in rat islets and insulin action in mouse hepatocytes [12,13]. Mitochondrial functionality directly translates into the functionality of the entire cell, including insulin signal transduction and thus insulin sensitivity. With the excessive supply of free fatty acids seen in obesity, there is an increase in the rate of mitochondrial metabolism, and prolonged exposure of cells to excess nutrients leads to mitochondrial overload. This mitochondrial dysfunction can contribute to chronic oxidative stress, which plays a significant role in the pathogenesis of insulin resistance in adipocytes [14,15,16]. Restoring normal mitochondrial function can have a therapeutic effect.

In our previous paper, we reported the therapeutic effect of cinnamic acid and its derivatives on insulin-stimulated glucose uptake (ISGU) in insulin-resistant 3T3-L1 adipocytes [17]. However, trying to elucidate the possible mechanism of action through which these compounds increase insulin sensitivity, we did not observe any changes in the expression of insulin pathway genes in treated cells compared to untreated cells. For this reason, we further examined the possible mechanisms through which these compounds overcome insulin resistance. Considering the significant role of mitochondria in overall cell metabolism, we hypothesized that the mechanism might directly involve mitochondria. To verify this assumption, we investigated the number of mitochondria and the expression of genes encoded by mtDNA in insulin-resistant adipocytes treated with the examined substances to identify a potential mechanism underlying increased cellular sensitivity to insulin.

## 2. Materials and Methods

### 2.1. Study Reagents

Cinnamic acid (CA) was purchased from Sigma Aldrich (Saint Louis, MO, USA). The stock solution was prepared by dissolving it in 1 mL of ethanol, obtaining a 235 mM concentration of working solution, and it was further diluted in cell culture media to the experimental concentrations (25 μM, 50 μM, and 125 μM).

**Figure 1:**
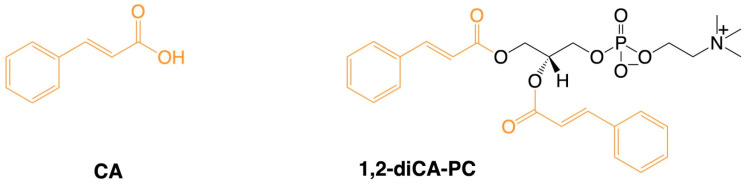
Chemical structure of tested compounds. CA−cinnamic acid, 1,2-diCA-PC−1,2-dicinnamoyl-*sn*-glycero-3-phosphocholine.

1,2-Dicinnamoyl-*sn*-glycero-3-phosphocholine (1,2-diCA-PC) was synthesized in the Department of Food Chemistry and Biocatalysis at Wrocław University of Environmental and Life Sciences, following the previously reported procedure [18,19]. CA was esterified with the cadmium complex of *sn*-glycero-3-phosphocholine in the presence of 4-(*N*,*N*-dimethylamino)pyridine (DMAP) as a catalyst and *N*,*N*′-dicyclohexylcarbodiimide (DCC) as a coupling agent. After purification, the pure product stock solution was prepared by dissolving it in 1 mL of DMSO, obtaining a 44 mM concentration of working solution, and then it was further diluted in cell culture media to the experimental concentrations (25 μM, 50 μM, and 125 μM). Figure 1 illustrates the chemical structures of the analyzed compounds.

### 2.2. The 3T3-L1 Cell Line Culturing and Differentiation

Mouse fibroblasts 3T3-L1 were purchased from ATCC (Manassas, VA, USA, CL-173™). Cells were cultured in DMEM (Dulbecco’s Modified Eagle’s Medium, 4.5 g/L of glucose, Corning Incorporated, New York, NY, USA) supplemented with 10% fetal calf serum (FCS, Sigma-Aldrich, Saint Louis, MI, USA) and antibiotics (penicillin, 50 U/mL; streptomycin, 50 µg/mL, Corning Incorporated, New York, NY, USA) in a humidified incubator at 37 °C and with 5% CO₂.

After achieving 100% confluence, differentiation into mature adipocytes was induced by changing the medium to DMEM (4.5 g/L of glucose) supplemented with 10% fetal bovine serum (FBS, Corning Incorporated, New York, NY, USA), antibiotics (penicillin, 50 U/mL; streptomycin, 50 µg/mL), 3-isobutyl-1-methylxanthine (115 µg/mL), dexamethasone (390 ng/mL), and insulin (10 µg/mL). After three days, the medium was changed to DMEM (4.5 g/L of glucose) with antibiotics, 10% FBS, and insulin (10 µg/mL). After an additional three days, the medium was changed to DMEM (4.5 g/L of glucose) with antibiotics and 10% FBS, and the cells were further cultured for an additional two days. The cells reached maturity 8 days after the initiation of differentiation.

### 2.3. Insulin Resistance Induction and the Effect of Phospholipid Derivatives

Insulin resistance was induced in mature adipocytes by culturing the cells in DMEM (4.5 g/L of glucose) supplemented with 10% FBS, antibiotics (penicillin, 50 U/mL; streptomycin, 50 µg/mL), and palmitic acid 16:0 (Sigma-Aldrich, Saint Louis, MO, USA) at a concentration of 0.5 mM for 48 h [9].

After inducing insulin resistance, the analyzed compounds, cinnamic acid (CA) and 1,2-dicinnamoyl-*sn*-glycero-3-phosphocholine (1,2-diCA-PC), were added at three various concentrations (25 μM, 50 μM, and 125 μM). The concentrations were chosen based on a viability test described previously [14]. The analyzed compounds were added to DMEM (4.5 g/L of glucose) supplemented with 10% FBS, antibiotics (penicillin, 50 U/mL; streptomycin, 50 µg/mL), and palmitic acid (0.5 mM). For the controls, ethanol and DMSO were added to a final concentration of 0.25% in cultured medium. The adipocytes were then further cultured for an additional 48 h. Palmitic acid was included in the culture medium to maintain insulin resistance in the adipocytes. After incubation with the specific compounds, the cells were evaluated for insulin responses using a glucose uptake test, measuring both basal glucose uptake (BGU) and insulin-stimulated glucose uptake (ISGU).

### 2.4. Glucose Uptake Test

The Glucose Uptake-Glo Assay (Promega Corporation, Madison, WI, USA) was performed after 48 h of incubation with cinnamic acid (CA) and 1,2-dicinnamoyl-sn-glycero-3-phosphocholine (1,2-diCA-PC). The experiment was performed in four replicates. Cells were plated, differentiated, and the glucose uptake test was carried out on a 96-well plate. The day before the glucose uptake test, cells were starved in serum-free culture medium overnight. Just before starting the experiment, the culture medium was discarded, and the cells were washed twice with PBS to remove any residual glucose. Next, a portion of the experimental cells was stimulated with 1 µM insulin in PBS for 10 min (INS+), while the remaining adipocytes were left unstimulated (INS−). After insulin stimulation, 10 mM of 2-deoxyglucose (2DG6P) was added to all cells, and they were further incubated for an additional 10 min to assess both basal glucose uptake and insulin-stimulated glucose uptake. The cells were then processed according to the manufacturer’s protocol. Luminescence was measured using the Victor3, 1420 Multilabel Plate Reader from PerkinElmer.

### 2.5. DNA and RNA Extraction from Experimental and Control Cells

DNA from the cells was isolated using the phenol/chloroform method (Sigma-Aldrich, St. Louis, MO, USA). Total RNA from the cells was isolated using the trizol method (Sigma-Aldrich, St. Louis, MO, USA). DNA and RNA concentration was measured using a NanoDrop 2000 spectrophotometer (Thermo Scientific™, Waltham, MA, USA).

RNA samples were treated with DNase before the reverse transcription reaction using RNase-Free DNase (Promega Corporation, Madison, WI, USA) according to the manufacturer’s protocol. The experiment was performed in triplicate, and cells were cultured on 6-well plates.

### 2.6. Mitochondrial DNA Copy Number Quantification

Mitochondrial DNA copy numbers were quantified using the Absolute Mouse Mitochondrial DNA Copy Number Quantification qPCR Assay Kit (ScienCell™ Research Laboratories, Carlsbad, CA, USA). Procedures and calculations were performed according to the manufacturer’s protocol. Real-time PCR was used to quantify mtDNA and gDNA of the analyzed samples and reference human genomic DNA using the known mtDNA copy number quantified per diploid cell. The mtDNA copy number was calculated based on Cq values using the ∆∆Cq formula in reference to gDNA using SCR primers, provided by the producer. Next, the mtDNA was quantified by normalization to quantified standard concentration provided by the producer (724 copies ± 16 per diploid cell) using the following formulas:

∆∆Cq = ∆Cq (mtDNA) − ∆Cq (SCR),(1)

where SCR represents primers specific for gDNA.

mtDNA copy number = (724 ± 16) × 2^−∆∆Cq^, (2)

where (724 ± 16) is the standard concentration.

### 2.7. cDNA Synthesis and Quantification of Gene Expression

Reverse transcription was performed using the High-Capacity cDNA Reverse Transcription Kit (Applied Biosystems, Waltham, MA, USA). A total of 200 ng of isolated RNA was used for cDNA synthesis. Gene expression analysis was conducted by real-time PCR using Fast SYBR Green Master Mix (Applied Biosystems, Waltham, MA, USA). Primers for mtDNA-encoded genes—*Nd3* (NADH dehydrogenase subunit 3), *Nd4* (NADH dehydrogenase subunit 4), *Nd5* (NADH dehydrogenase subunit 5), *Cytb* (cytochrome b), *Atp6* (ATP synthase 6), *Atp8* (ATP synthase 8), *Cox1* (cytochrome c oxidase 1), and *Cox2* (cytochrome c oxidase 2)—as well as for the nuclear DNA (nDNA)-encoded reference gene *β-actin* (beta-actin) were manually designed (Table 1).

The specificity of the primers was checked using Primer v.3-BLAST (NCBI), and secondary structures were analyzed using OligoAnalyzer (Integrated DNA Technology). The acceptance criteria were DeltaG > −7 kcal/mol. Prior to real-time PCR, the efficiency of the primers was assessed using the standard curve method. Specificity was further confirmed based on the melting curve. Only primers with efficiency values higher than R^2^ ≥ 0.95 and confirmed specificity through the melting curve were used for gene expression studies. Relative gene expression levels, normalized to the housekeeping gene *β-actin*, were calculated using the delta–delta Ct (ΔΔCt) method. The fold change was determined using the 2^(−ΔΔCt)^ algorithm.

### 2.8. Statistical Analysis

Statistical analyses were performed using Microsoft Office Excel 2007 and Statistica 13.1 (StatSoft, Tulsa, OK, USA). The normality of the distribution was assessed using the Shapiro–Wilk test. Based on the results, parametric tests (Student’s *t*-test) were used for statistical calculations and analysis of differences between the studied groups. The results show the mean ± SD, and the number of repeats (*n*) is specified in the legends. Statistical significance was set at *p* < 0.05.

## 3. Results

### 3.1. Measurements of Glucose Uptake by Insulin-Resistant 3T3-L1 Adipocytes Cultured with Analyzed Compounds

**Figure 2:**
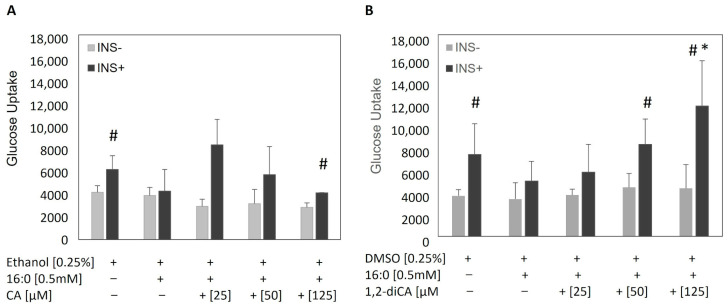
Glucose uptake in insulin-resistant 3T3-L1 adipocytes treated with the analyzed compounds (**A**) CA (cinnamic acid) and (**B**) 1,2-diCA-PC (1,2-dicinnamoyl-*sn*-glycero-3-phosphocholine) at concentrations of 25 µM, 50 µM, and 125 µM. INS− represents basal glucose uptake, and INS+ represents insulin-stimulated glucose uptake. Two controls were included: insulin-sensitive cells not treated with 16:0 and insulin-resistant cells treated with 16:0. * *p* < 0.05 indicates a statistical difference between experimental cells and IR adipocytes; # *p* < 0.05 indicates a statistical difference between ISGU and BGU. *n* = 4 repeats.

First of all, we tried to determine if the analyzed compounds at the working concentrations were able to abolish insulin resistance in 3T3-L1 adipocytes. The experiment was conducted in four repetitions. As a reference control, insulin-resistant cells incubated with the appropriate solvent (ethanol and DMSO for CA and 1,2-diCA-PC dissolving, respectively) were used. In our study, we did not detect any influence of ethanol or DMSO on insulin-stimulated glucose uptake by insulin-sensitive (IR−) and insulin-resistant (IR+) adipocytes. Cells with developed insulin resistance (IR+ ethanol, IR + DMSO) showed only a slight increase in ISGU in insulin-treated cells (INS+) compared to BGU (INS−). On the other hand, adipocytes with proper insulin sensitivity (IR—ethanol, IR—DMSO) displayed significantly increased ISGU compared to BGU (IR—ethanol, *p* = 0.024; IR—DMSO, *p* = 0.043; Figure 2).

Cinnamic acid stimulated insulin-stimulated glucose uptake at all studied concentrations compared to basal glucose uptake. The increase was 2.3-fold for 25 μM, 2-fold for 50 μM, and 1.6-fold for 125 μM (25 μM: *p* = 0.062; 50 μM: *p* = 0.093; 125 μM: *p* = 0.009, Figure 2); however, the increase was only statistically significant for 125 μM. When compared to the insulin-stimulated insulin-resistant adipocytes (INS+, IR+), we did not observe any significant changes in glucose uptake in IR adipocytes treated or not treated with CA, and the difference was not noticeable at any of the analyzed concentrations.

The 1,2-diCA-PC significantly increased ISGU at the two highest concentrations in cells stimulated by insulin compared to nonstimulated cells. For the concentrations of 50 μM and 125 μM, the increase was 2.9-fold and 3.5-fold, respectively (50 μM: *p* = 0.047; 125 μM: *p* = 0.013, Figure 2), showing a dose-dependent effect. When comparing the ISGU in adipocytes treated with 1,2-diCA-PC to insulin-resistant adipocytes (IR+), we observed a significant increase in glucose uptake at the highest concentration, which was almost three times higher (*p* = 0.034). The medium concentration of 1,2-diCA-PC (50 μM) increased ISGU by about two times (*p* = 0.108). The lowest concentration of 1,2-diCA-PC (25 μM) did not increase ISGU in insulin-resistant adipocytes when compared to IR adipocytes not treated with this compound.

### 3.2. Analysis of mtDNA Copy Number in Experimental Cells Treated with Analyzed Compounds

After confirming the effect of analyzed compounds on insulin signaling and glucose uptake, next, we wanted to verify if the mechanism of action of analyzed compounds influenced mitochondria metabolism. Thus, first, we evaluated the number of mitochondria by mtDNA quantification. The mtDNA copy numbers obtained for experimental cells treated with CA and 1,2-diCA-PC at appropriate concentrations were compared to control insulin-resistant adipocytes (IR+) as well as to adipocytes with proper insulin sensitivity (IR−) incubated solely with an appropriate solvent (ethanol and DMSO).

**Figure 3:**
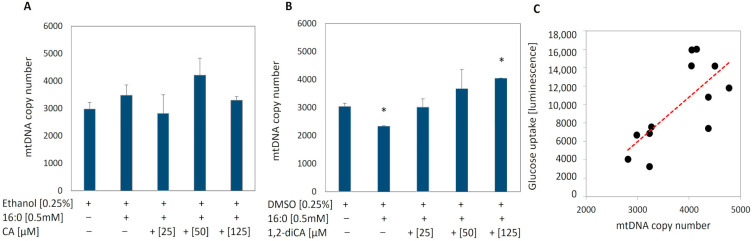
mtDNA copy number assessed by real-time PCR in insulin-resistant adipocytes with proper insulin sensitivity (not treated with 16:0) and with induced insulin resistance (treated with 16:0), as well as in adipocytes treated with (**A**) cinnamic acid (CA) and (**B**) 1,2-diCA-PC at appropriate concentrations (25 µM, 50 µM, and 125 µM). (**C**) The correlation between mtDNA copy number and ISGU observed for 1,2-diCA-PC (125 µM), * *p* < 0.05. *n* = 3 repeats.

First, we focused on the evaluation of mtDNA copy number between insulin-resistant and insulin-sensitive adipocytes. In cells where ethanol was used as the solvent, we observed a slightly higher amount of mtDNA in control cells with induced insulin resistance (IR+ ethanol) compared to cells with normal insulin sensitivity (IR− ethanol); however, the increase was not statistically significant. On the other hand, we noticed a statistically significant reduction in the amount of mtDNA in insulin-resistant adipocytes (IR+ DMSO) compared to cells with normal insulin sensitivity (IR− DMSO) (*p* = 0.013) (Figure 3A,B).

No substantial differences in the mtDNA copy number were observed in experimental cells treated with CA at any of the tested concentrations when compared to IR+ cells (Figure 3A).

In adipocytes treated with the 1,2-diCA-PC, we observed a significant increase in the amount of mtDNA at all tested concentrations in a dose-dependent manner. The largest increase, up to 73% compared to the control adipocytes (IR+), was observed at a concentration of 125 μM (*p* = 0.000), followed by a 57% increase at 50 μM (*p* = 0.078) and a 29% increase at 25 μM (*p* = 0.081, Figure 3B).

We also searched for any correlation between glucose uptake rate after insulin stimulation (ISGU) and mtDNA copy number. We noticed a strong correlation between mtDNA copy number and ISGU in insulin-resistant adipocytes treated with 1,2-diCA-PC (R = 0.71, *p* = 0.008; Figure 3C). No statistically significant correlation has been observed for cinnamic acid.

### 3.3. The Measurements of Expression of mtDNA-Encoded Genes in Experimental Cells Treated with Analyzed Compounds

**Figure 4:**
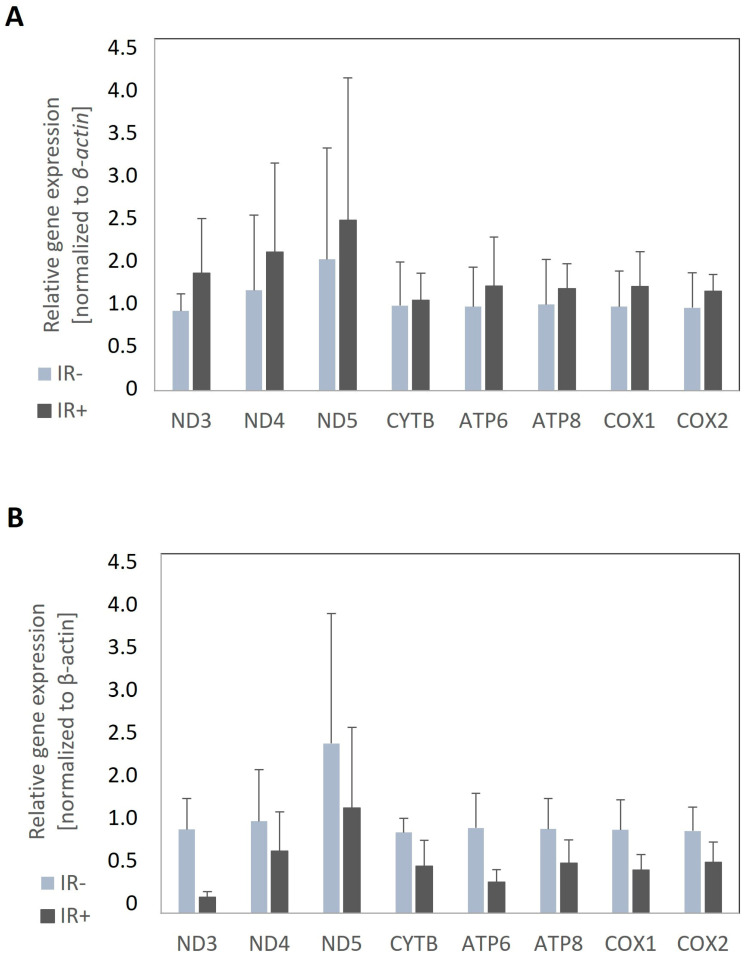
The expression of genes encoded by mtDNA in control adipocytes: IR− cells (insulin-sensitive, not treated with 16:0) and IR+ cells (insulin-resistant, treated with 16:0). The analysis was conducted for two types of control cells: those treated with ethanol (**A**) and those treated with DMSO (**B**). *n* = 3 repeats.

Besides the number of mitochondria, we also evaluated the expression of mtDNA-encoded genes as part of the mitochondrial function. Similar to mtDNA copy number determination, first, the differences between control adipocytes with induced insulin resistance (IR+) and insulin-sensitive (IR−) adipocytes cultured with the appropriate solvent were evaluated to detect any differences in mtDNA-encoded gene expression. In other words, we aimed to determine if insulin resistance caused any changes in the expression of mitochondrial genes. The expression levels of all analyzed genes were markedly reduced in IR adipocytes compared to insulin-sensitive control cells. Surprisingly, the downregulation of mitochondrial-encoded genes was observed only in cells cultured with DMSO (used as the solvent). In control cells treated with ethanol, we did not observe a reduction in expression rates (Figure 4A). It should also be noted that the reduction in expression rates in cells cultured with DMSO was not statistically significant, although the downregulation of several genes approached significance (Nd3 *p* = 0.063; Cytb *p* = 0.118; Atp6 *p* = 0.063; Atp8 *p* = 0.197; Cox1 *p* = 0.110; Cox2 *p* = 0.158, Figure 4B).

**Figure 5:**
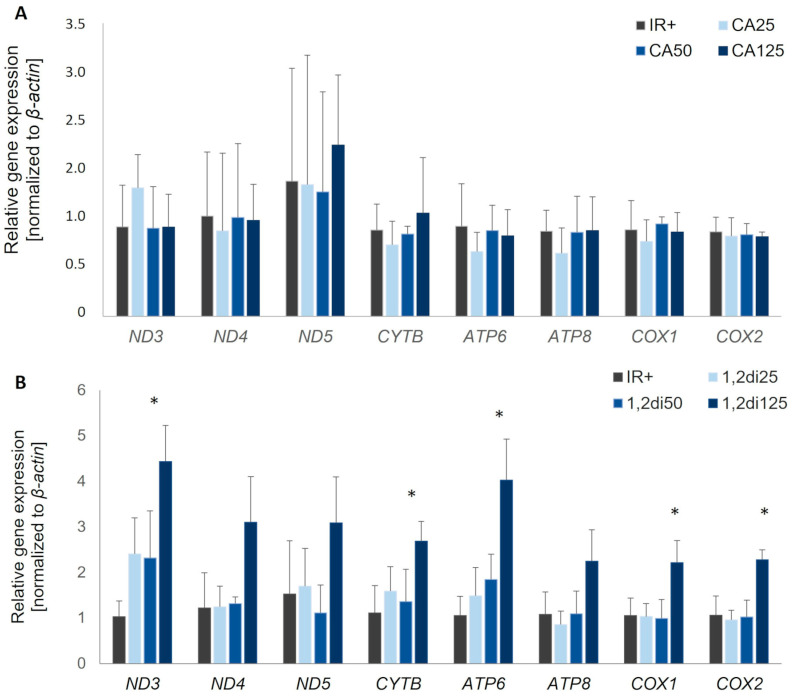
The expression of mitochondrial genes in insulin-resistant adipocytes treated with (**A**) cinnamic acid at three concentrations (25 µM, 50 µM, and 125 µM) compared to insulin-resistant control adipocytes (IR+); (**B**) 1,2-diCA-PC at three concentrations (25 µM, 50 µM, and 125 µM) compared to insulin-resistant control adipocytes (IR+). * *p* < 0.05. *n* = 3 repeats.

Next, we verified whether cinnamic acid or its derivative 1,2-diCA-PC influence mtDNA-encoded gene expression. Cinnamic acid did not influence the expression of genes encoded by mtDNA in experimental cells compared to insulin-resistant adipocytes (IR+). Cells incubated with CA at three tested concentrations (25 μM, 50 μM, and 125 μM) did not exhibit any significant changes in the expression levels of almost all analyzed genes (Figure 5A). Opposite to CA, insulin-resistant adipocytes treated with 1,2-diCA-PC exhibited a significant increase in the expression of all analyzed genes compared to insulin-resistant cells (IR+). However, the increase was notable only at the concentration of 125 μM of 1,2-diCA-PC. We observed the upregulation of the following genes: a 4.3-fold increase for Nd3 (*p* = 0.017), a 3.8-fold increase for Atp6 (*p* = 0.022), and an approximately 2-fold increase for Nd4 (*p* = 0.106), Nd5 (no statistical significance), Cytb (*p* = 0.021), Atp8 (*p* = 0.134), Cox1 (*p* = 0.050), and Cox2 (*p* = 0.022) (Figure 5B). Cells treated with lower concentrations of 1,2-diCA-PC (25 and 50 μM) displayed a slight increase in expression for Nd3, Cytb, and Atp6 only, but these changes were not statistically significant (Figure 5B).

## 4. Discussion

In the present study, we evaluated the influence of cinnamic acid and its phospholipid derivative, 1,2-dicinnamoyl-sn-glycero-3-phosphocholine (1,2-diCA-PC), on mitochondrial function in insulin-resistant 3T3-L1 adipocytes by investigating the number of mitochondria and the expression of genes encoded by mtDNA.

Firstly, we confirmed the successful development of insulin resistance in mature adipocytes using palmitic acid (16:0), assessed by insulin-stimulated glucose uptake. We observed a lower glucose uptake rate in cells treated with palmitic acid in both types of insulin-resistant controls—those incubated with ethanol or DMSO, the solvents used for resuspending CA and 1,2-diCA-PC, respectively—compared to cells where insulin resistance was not induced.

Next, after incubation with the examined compounds, we performed insulin-stimulated glucose uptake measurements in insulin-resistant adipocytes. For both analyzed compounds, CA and 1,2-diCA-PC, we confirmed previous observations that CA and 1,2-diCA-PC increase insulin-stimulated glucose uptake in adipocytes with previously developed insulin resistance [17]. It was previously reported that CA moderately increased glucose uptake in insulin-resistant adipocytes, while 1,2-diCA-PC significantly increased the glucose uptake rate, effectively doubling the level of glucose uptake in insulin-sensitive adipocytes [17]. However, in the present study, we used a lower concentration of analyzed compounds in order to investigate dose dependency. Despite the variance, the observation obtained previously, but for higher concentrations, was replicated. As the possible mechanisms of action had not been previously revealed, we further investigated how cinnamic acid and its derivative could enhance glucose uptake and restore proper insulin signaling.

The number and size of mitochondria are crucial for the proper functioning of these organelles, and they are correlated with mitochondrial oxidative capacity and energy efficiency [20]. A reduced content of mtDNA has been observed in blood cells of obese subjects. Studies have demonstrated a strong negative correlation between mtDNA content and BMI, as well as between mtDNA content and the amount of visceral fat [21]. Furthermore, a reduced amount of mtDNA has also been observed in obese subjects with concomitant insulin resistance. The mtDNA/nDNA (nuclear DNA) ratio was inversely correlated with HOMA, glucose, and uric acid levels [22,23]. Additionally, in obese subjects with diagnosed T2D, a negative correlation was observed between mtDNA levels and BMI, fasting plasma glucose, fasting plasma insulin, LDL, and TG values in the blood [24]. A reduction in electron transport chain activity, which correlated with a reduced number of mitochondria, was observed in the skeletal muscle of obese individuals [25,26].

All these reports highlight the significant role of mitochondria not only in the pathogenesis of metabolic diseases but also in their interrelation. In our study, we observed a reduced number of mitochondria in insulin-resistant adipocytes compared to cells with proper insulin sensitivity. However, this decrease was observed only in cells cultured with DMSO and palmitic acid (used as the control for 1,2-diCA-PC). Slightly different results were obtained for adipocytes treated with palmitic acid and ethanol. In these cells, the number of mtDNA copies was even slightly higher in insulin-resistant adipocytes compared to adipocytes with proper insulin sensitivity.

Similarly, when comparing the expression of mitochondrial-encoded genes between insulin-resistant adipocytes and adipocytes with proper insulin sensitivity, we noticed downregulation in insulin-resistant adipocytes treated with palmitic acid and DMSO. However, no changes in expression or even slightly increased expression of mitochondrial-encoding genes were observed in adipocytes co-treated with palmitic acid and ethanol, suggesting some influence of ethanol on mitochondrial biogenesis. Further research is needed to elucidate the effects of ethanol on mitochondrial biogenesis, especially given that some of the literature provides evidence of ethanol’s negative impact on mitochondrial metabolism [27]. This may take time as most studies refer to chronic alcohol intake, whereas our ethanol stimulation lasted only 48 h.

Despite these observations, we can strongly conclude that our results support previous findings reported by others, confirming that mitochondrial numbers are decreased in insulin resistance. The influence of ethanol on mitochondrial biogenesis and metabolism certainly requires more in-depth research.

Similar to our previous report, we observed a positive effect of the examined compounds on insulin-stimulated glucose uptake in insulin-resistant adipocytes. However, similar to previous reports, the glucose uptake stimulated by cinnamic acid (CA) was moderate across all studied concentrations [9]. In contrast, the results obtained for the derivative 1,2-dicinnamoyl-sn-glycero-3-phosphocholine (1,2-diCA-PC) demonstrated a dose-dependent increase in insulin-stimulated glucose uptake. At the highest tested concentration, 1,2-diCA-PC doubled the glucose uptake rate measured in adipocytes with proper insulin sensitivity. Similarly, we observed a dose-dependent increase in the number of mitochondria in insulin-resistant cells treated with 1,2-diCA-PC at all analyzed concentrations (25 μM, 50 μM, and 125 μM) compared to control cells without 1,2-diCA-PC. Moreover, we observed a positive correlation between the number of mtDNA copies and insulin-stimulated glucose uptake in adipocytes treated with 1,2-diCA-PC at the concentration of 125 μM, which supports the effect of increased mitochondrial copy number on enhancing cellular sensitivity to insulin.

To further study the relationship between mitochondria and insulin sensitivity, we measured the expression rate of genes encoded by mtDNA in experimental cells. The mtDNA genes encode proteins that are crucial for the enzymatic activity of the electron transport chain; thus, the regulation of these genes is vital for the proper functioning of cellular metabolism. A decreased expression of *Nd1* and *Cox2* has been observed in cells from Zucker diabetic fatty (ZDF) rats, an animal model of type 2 diabetes, compared to lean animals. Moreover, the expression levels of these genes are inversely correlated with plasma glucose levels [28]. A 50% decrease in the expression of *Cox1*, a subunit of mitochondrial complex IV, has been observed in insulin-resistant offspring of T2D parents [29]. In our study, we observed a significant increase in the expression levels of all analyzed mtDNA-encoded genes in cells treated with 1,2-diCA-PC at a concentration of 125 μM compared to control cells.

The results described above suggest a potential mechanism by which 1,2-diCA-PC, at a concentration of 125 μM, reverses insulin resistance in adipocytes by enhancing mitochondrial biogenesis, as indicated by increased mtDNA copy numbers. A comprehensive understanding of 1,2-diCA-PC’s effect on reversing insulin resistance can be gained by considering the results of gene expression, where the highest tested concentration significantly increased the expression of numerous mtDNA-encoded genes.

Cinnamic acid and its numerous derivatives have a positive effect on carbohydrate metabolism by regulating lipid metabolism. In general these compounds are considered as antioxidant and anti-inflammatory agents, which improve the antioxidant status by reducing oxidative stress in patients, mostly influencing the LDL oxidation rate [30]. It has been shown that cinnamic acid and its derivatives lower plasma and liver triglyceride and cholesterol levels by reducing the HMG-CoA reductase and ACAT activity in experimental animals, thus influencing the FFA level [31,32]. FFA level has both positive and negative effects on mitochondria content and metabolism. FFAs are essential dietary nutrients; however, when present in excess, they inhibit mitochondrial biogenesis and function, mainly via the PPARα pathway which activates the uncoupling protein (UCP) [33]. This might be another aspect that should be taken into account during further evaluation of cinnamic acid’s effect on mitochondria metabolism, especially in terms of 1,2-diCA-PC.

In 3T3-L1 adipocytes with developed insulin resistance, treatment with 1,2-diCA-PC at 125 μM led to increased glucose uptake, indicating improved cellular sensitivity to insulin. This effect was accompanied by a significant increase in the number of mitochondria and the expression levels of all analyzed mtDNA-encoded genes compared to control cells. Our studies demonstrate a relationship between improved cellular insulin sensitivity and an increase in both mitochondrial numbers and mtDNA-encoded gene expression. All analyzed mtDNA genes encode proteins that are components of the electron transport chain and are essential for oxidative phosphorylation. Furthermore, these results highlight the significant therapeutic potential of the cinnamic acid derivative 1,2-diCA-PC in enhancing the insulin sensitivity of adipocytes.

The results presented also have some limitations. The most significant limitation of this study is the lack of a detailed molecular mechanism explaining how 1,2-diCA-PC enhances mitochondrial biogenesis, improves insulin response, and reverses insulin resistance. Further research is needed to fully elucidate this mechanism. Additionally, it would be valuable to investigate whether similar effects are observed in other insulin-dependent cells, such as skeletal muscle cells or hepatocytes, and to explore if higher concentrations of 1,2-diCA-PC might produce stronger effects. Moreover, the expression of mtDNA-encoded genes could be further validated by quantifying protein levels using Western blot analysis. Finally, the effects of expression rates in control cells treated with solvents alone yielded various results. A significantly lower expression of mtDNA-encoded genes was observed in insulin-resistant cells treated with DMSO compared to insulin-sensitive controls, with no changes in the expression of those genes in control cells where ethanol was used. Similarly, further exploration of this effect would provide additional insight into mitochondrial metabolism. Despite these limitations, this study is the first to report that 1,2-diCA-PC can reverse insulin resistance by increasing mtDNA copy number and upregulating key genes involved in the enzymatic complexes of oxidative phosphorylation.

## 5. Conclusions

In the present study, we demonstrated changes in both mtDNA copy number and the expression of mitochondrial genes in adipocytes as possible mechanisms through which 1,2-diCA-PC enhances the insulin response. We concluded that 1,2-diCA-PC reverses insulin resistance by promoting mitochondrial biogenesis and upregulating mitochondrial gene expression, but this effect is only observed at concentrations above 125 μM.

## Figures and Tables

**Table 1:** The sequences of primers used for mitochondrial gene expression analysis.

Gene	Official Full Name of Gene	Primer’s Sequence (5′→3′)	R^2^ Value
*Nd3*	NADH dehydrogenase subunit 3	F *: TAACCTGTACACTGTTATCTTC	0.99
		R *: GAGTACAGATTTATTTGGGGG	
*Nd4*	NADH dehydrogenase subunit 4	F: TCCTCAGTTAGCCACATAGC	0.99
		R: ATAAGTGGGAAGACCATTTGAA	
*Nd5*	NADH dehydrogenase subunit 5	F: CCCATGACTACCATCAGCAA	0.99
		R: ATAATGTGGTTAGGGCTCCG	
*Cytb*	Cytochrome b	F: CATACGAAAAACACACCCATTA	0.99
		R: GTAGTGTATGGCTAAGAAAAGA	
*Atp6*	ATP synthase 6	F: TTTACACCTACTACCCAACTAT	0.99
		R: GGAATTAGTGAAATTGGAGTTC	
*Atp8*	ATP synthase 8	F: GCCACAACTAGATACATCAAC	0.98
		R: GAAGGTGCCAGTGGGAATG	
*Cox1*	Cytochrome c oxidase 1	F: TATCTACTATTCGGAGCCTGA	0.99
		R: GCATGGGCAGTTACGATAAC	
*Cox2*	Cytochrome c oxidase 2	F: GAAGACCTATGCTTTGATTCAT	0.99
		R: GGATTGGAAGTTCTATTGGCA	
*Β-actin*	Beta-actin	F: CGTTGACATCCGTAAAGACC	0.99
		R: CTAGGAGCCAGAGCAGTAAT	

* F: forward primer; R: reverse primer.

## Data Availability

The original contributions presented in the study are included in the article; further inquiries can be directed to the corresponding authors.
